# Supplementary material for: Audiovisual integration in macaque face patch neurons
Source: Curr Biol. 2021 May 10;31(9):1826–1835.e3. doi: 10.1016/j.cub.2021.01.102 (PMC8521527; doi:10.1016/j.cub.2021.01.102)
Supplement: Document S1. Figures S1–S3 [file mmc1.pdf]

Current Biology, Volume *31*

Supplemental Information

Audiovisual integration

in macaque face patch neurons

Amit P. Khandhadia, Aidan P. Murphy, Lizabeth M. Romanski, Jennifer K. Bizley, and David A. Leopold

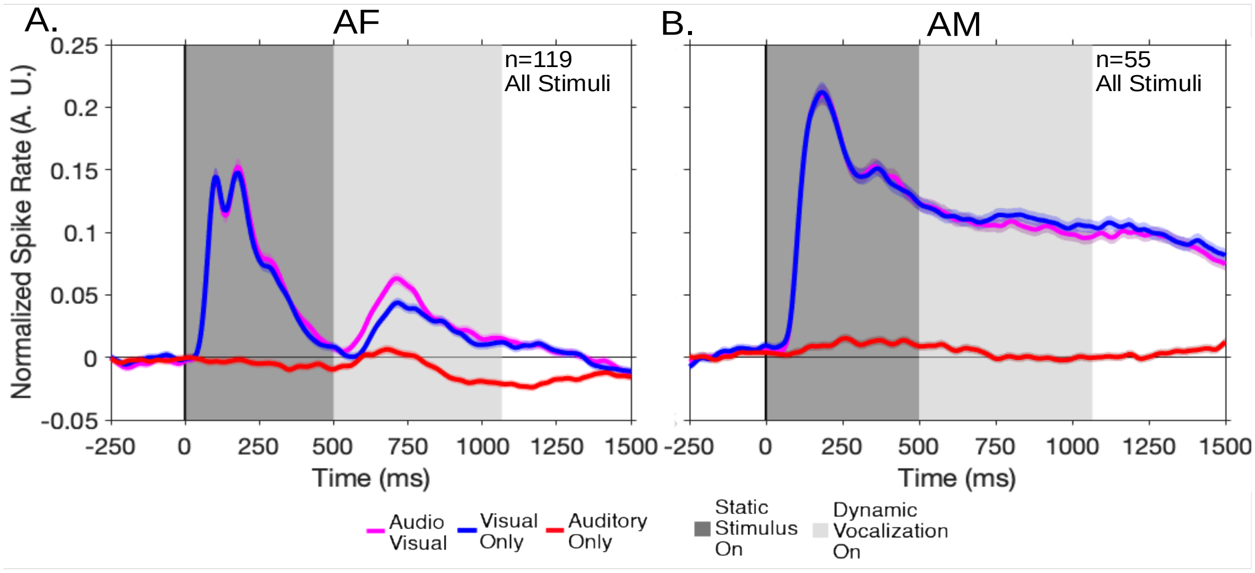

Figure S1. Grand average responses across all cells and all stimuli for face patches, related to Figure 2. A, B SDF plots of the grand average for AF, AM respectively (the SEM for each response is shown in a cloud surrounding each line with a very low SEM for AF)

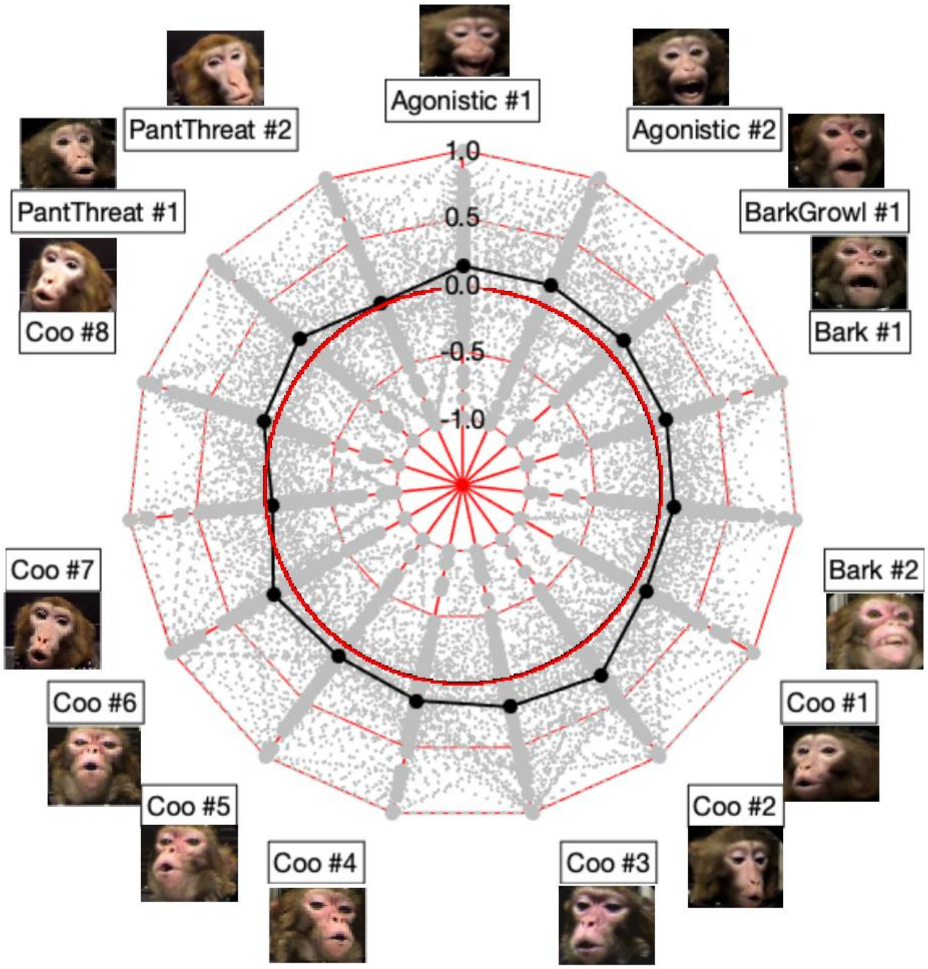

Figure S2 Effects of different stimuli on audiovisual integration, related to Figure 3. A radar plot showing the AV index for all cells in AF for each stimulus in the light gray with the mean represented in the black line and the zero mark in the red line.

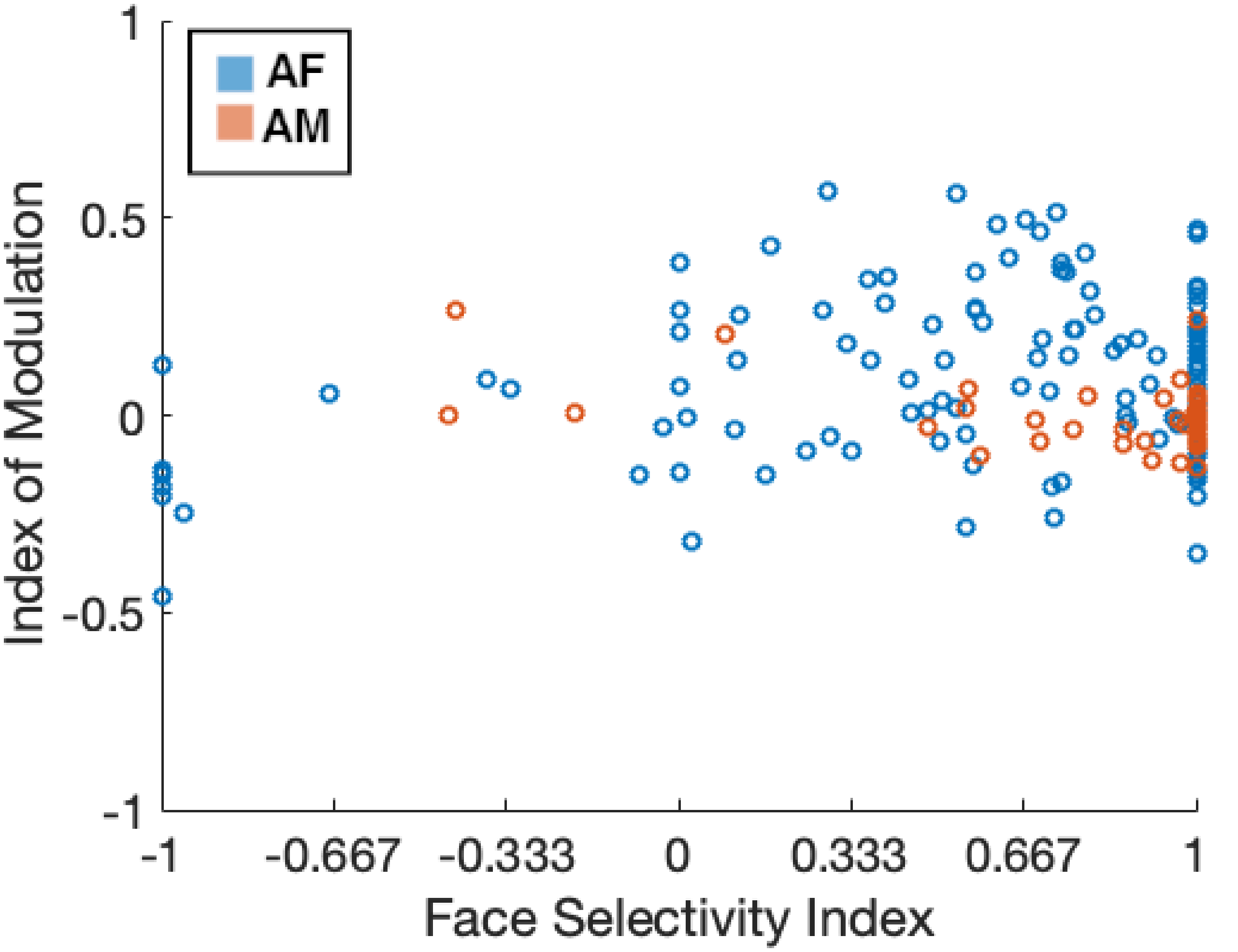

Figure S3. Relationship of Face Selectivity and Audiovisual Modulation related to Figure 3 and Table 1. Scatter plot comparing the face selectivity index with the index of modulation for all AF cells (blue) and AM cells (orange).
