# Supplementary material for: Audiovisual integration in macaque face patch neurons
Source: Curr Biol. 2021 May 10;31(9):1826–1835.e3. doi: 10.1016/j.cub.2021.01.102 (PMC8521527; doi:10.1016/j.cub.2021.01.102)
Supplement: Document S2. Article plus supplemental information [file mmc2.pdf]

Current Biology

Article

Audiovisual integration in macaque face patch neurons

Graphical Abstract

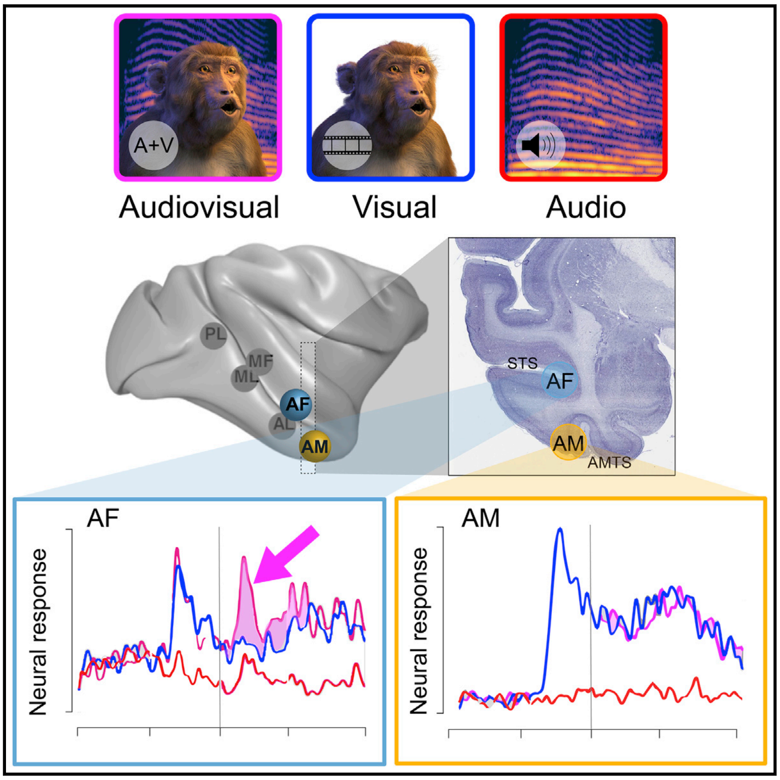

Authors

Amit P. Khandhadia, Aidan P. Murphy, Lizabeth M. Romanski, Jennifer K. Bizley, David A. Leopold

Correspondence

amit.khandhadia@nih.gov (A.P.K.),
leopoldd@mail.nih.gov (D.A.L.)

In Brief

Khandhadia et al. examine neural responses in two macaque face patches (AF and AM) to audiovisual renditions of vocalizing macaques. They find that the auditory component consistently modulates face-selective, single-unit activity in AF, but not in AM, suggesting an important role for AF in the audiovisual integration of social stimuli.

Highlights

- Audiovisual integration was examined among neurons in macaque AF and AM face patches
- Most neurons in AF were modulated by the acoustic component of macaque vocalizations
- Acoustic modulation in AF was contingent on visual facial structure
- Very few neurons in AM exhibited auditory responses or modulation

Khandhadia et al., 2021, Current Biology *31*, 1826–1835
May 10, 2021 Published by Elsevier Inc.
https://doi.org/10.1016/j.cub.2021.01.102

CellPress

CellPress
OPEN ACCESS

Current Biology

Article

Audiovisual integration in macaque face patch neurons

Amit P. Khandhadia,[1,2,*] Aidan P. Murphy,[1,3] Lizabeth M. Romanski,[4] Jennifer K. Bizley,[2] and David A. Leopold[1,3,5,*]
[1]Laboratory of Neuropsychology, National Institute of Mental Health, NIH, Bethesda, MD 20892, USA
[2]Ear Institute, University College London, 332 Gray's Inn Road, London WC1X 8EE, UK
[3]Neurophysiology Imaging Facility, National Institute of Mental Health, National Institute of Neurological Disorders and Stroke, National Eye Institute, NIH, Bethesda, MD 20892, USA
[4]Department of Neuroscience, University of Rochester School of Medicine, Rochester, NY 14642, USA
[5]Lead contact
*Correspondence: amit.khandhadia@nih.gov (A.P.K.), leopoldd@mail.nih.gov (D.A.L.)
https://doi.org/10.1016/j.cub.2021.01.102

SUMMARY

Primate social communication depends on the perceptual integration of visual and auditory cues, reflected in the multimodal mixing of sensory signals in certain cortical areas. The macaque cortical face patch network, identified through visual, face-selective responses measured with fMRI, is assumed to contribute to visual social interactions. However, whether face patch neurons are also influenced by acoustic information, such as the auditory component of a natural vocalization, remains unknown. Here, we recorded single-unit activity in the anterior fundus (AF) face patch, in the superior temporal sulcus, and anterior medial (AM) face patch, on the undersurface of the temporal lobe, in macaques presented with audiovisual, visual-only, and auditory-only renditions of natural movies of macaques vocalizing. The results revealed that 76% of neurons in face patch AF were significantly influenced by the auditory component of the movie, most often through enhancement of visual responses but sometimes in response to the auditory stimulus alone. By contrast, few neurons in face patch AM exhibited significant auditory responses or modulation. Control experiments in AF used an animated macaque avatar to demonstrate, first, that the structural elements of the face were often essential for audiovisual modulation and, second, that the temporal modulation of the acoustic stimulus was more important than its frequency spectrum. Together, these results identify a striking contrast between two face patches and specifically identify AF as playing a potential role in the integration of audiovisual cues during natural modes of social communication.

INTRODUCTION

In humans and other primate species, audiovisual integration plays an important role in social communication, for example, during the perception of a conspecific's vocalization and concomitant facial behavior.[1,2] The temporal cortex, and particularly the superior temporal sulcus (STS), contain zones of convergence for high-level sensory signals.[3–6] In the macaque, the STS fundus borders high-level visual and auditory cortex[7–10] and exchanges connections with other multisensory areas, including ventrolateral prefrontal cortex (VLPFC) and intraparietal cortex.[8,11,12] At the single-cell level, neurons within portions of the STS respond to visual and auditory stimuli, as well as their combination.[5,13–16] Functional MRI (fMRI) investigation of the macaque temporal cortex has also revealed a number of operationally defined regions named according to their visual category selectivity, such as face and body patches.[17–21] In macaques, face patches are replete with cells that respond more strongly to faces than to other categories of images[22–24] and form an interconnected network.[25,26] A subset of these patches lies along the STS and is coextensive with known multisensory regions in the fundus.[5,15] However, despite intensive study of neurons within the visually defined face patches, it is presently unknown whether or not they participate in multisensory integration.

Here, we investigated audiovisual single-unit responses in two fMRI-defined face patches. The anterior fundus (AF) and anterior medial (AM) patches were selected as key candidate regions for investigation, as they are both thought to occupy high-level positions in the face-processing hierarchy but are situated in distinct portions of the temporal cortex.[21,27] Area AF is located in the STS fundus, within regions known to contain multisensory neurons although AM is located on the undersurface of the temporal lobe surface adjacent to, and interconnected with, the perirhinal and parahippocampal cortices, which also receive multisensory information.[26,28] After identifying these patches based on their selective visual fMRI responses to faces, we recorded the activity of individual neurons within each patch to brief movie clips of macaque vocalizations, including the full audiovisual stimulus as well as the visual and auditory components alone. The results demonstrate that auditory information prominently influences the responses of AF neurons but has little effect on the responses of AM neurons. We then further evaluated the audiovisual modulation in AF with control experiments. These

This is an open access article under the CC BY license (http://creativecommons.org/licenses/by/4.0/).

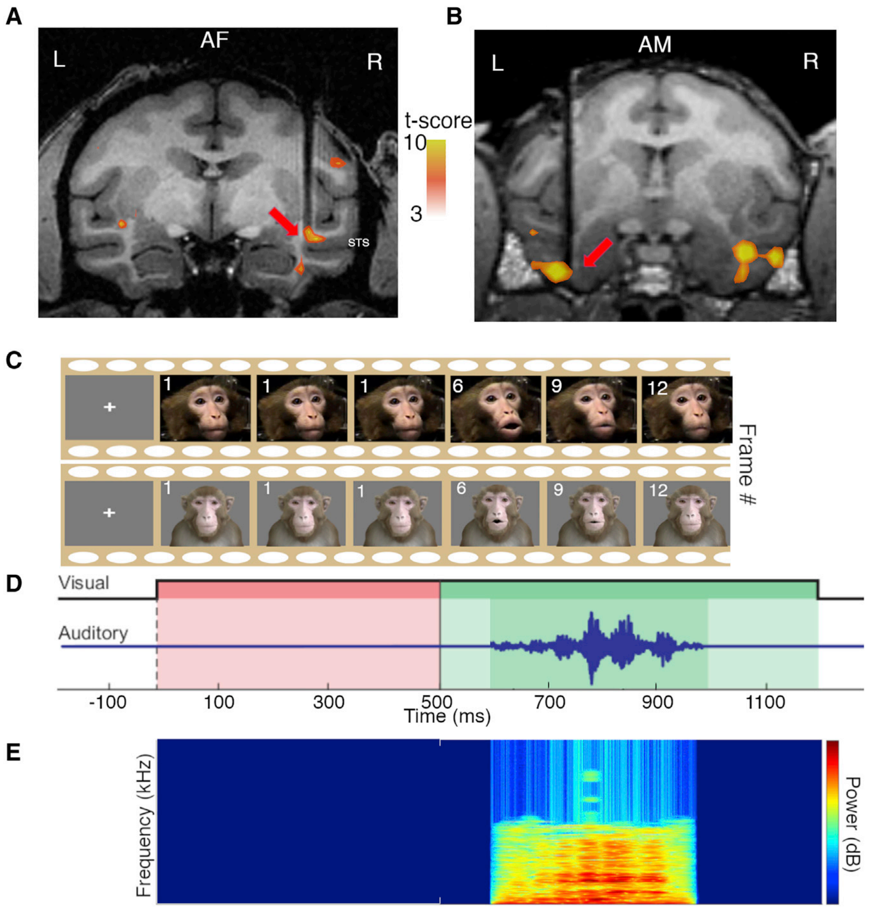

Figure 1. Localization of recording sites in the STS and IT cortex

(A and B) Functional overlays of AF and AM from monkey SP and monkey W, respectively, of an fMRI contrast of faces versus objects. The tract of the electrode is indicated with the red arrow targeted to the desired areas of recording.

(C) Pant-threat vocalization from an unfamiliar macaque.

(D) The same vocalization as performed by the avatar, both including the 500-ms still frame indicated by the frames labeled 1.

(E) Presentation timeline of stimulus indicating the onset of the still frame indicated in red and the onset of the movie in green as well as the auditory stimulus, including a spectrogram.

control experiments demonstrate that auditory modulation is specific to faces and depends on the temporal, rather than spectral, structure of the acoustic stimulus. We discuss the findings in relation to the layout of the macaque face patch network and its intersection with known audiovisual cortical areas.

RESULTS

We conducted extracellular recordings in fMRI-defined face patches in four adult macaque monkeys. Based on an initial fMRI mapping of face patches (see STAR methods), we targeted a single, chronic 64-channel microwire electrode bundle into the centers of the AF or AM face patches (Figures 1A and 1B). Each macaque received a single implant into a recorded face patch. We have previously demonstrated that this recording method supports longitudinal, stable recordings from the same cells over multiple sessions.[29,30] We recorded from 295 neurons in face patches of four monkey subjects: 240 from AF (125 from monkey SP, 115 from monkey SR) and 55 neurons from AM (49 from monkey W, 6 from monkey M). In addition to the main experimental conditions featured in the study, subjects viewed a short "fingerprinting" stimulus set of static images each day, which included human and monkey faces, objects, and scenes. This daily dataset allowed us both to determine each neuron's face selectivity index (see STAR methods) and to verify its identity across successive sessions.[29]

Consistent with previous studies, the majority of neurons in both AF and AM were face selective. Specifically, 84.1% of all neurons (198/240 of AF neurons and 50/55 of AM neurons) responded to flashed faces with a face selectivity index (FSI) absolute value of greater than 0.333, a criterion that has previously been used to categorize neurons as face selective,[22,24] and both face patches show a distribution of FSI greater than zero ($t_{(118)} = 11.375$, $p = 1 \times 10^{-20}$ for AF; $t_{(54)} = 11.702$, $p = 2 \times 10^{-16}$ for AM). During the main electrophysiological experiments, the animals were required to maintain their gaze anywhere within the visual stimulus or, in the case of auditory-only presentation, upon a small fixation marker. The dynamic component of the video was always preceded by a 500-ms static image of the face, corresponding to the first frame of the movie video. This presentation was incorporated to diminish the contribution of abrupt visual transients during the period of audiovisual integration under study (Figures 1C–1E). Subjects experienced 20–40 repetitions of each stimulus, receiving a juice reward after completion of each presentation.

Experiment 1: multisensory responses of AF and AM face patch neurons

The goal of the first experiment was to determine whether the addition of the auditory component of the vocalization influences the responses of neurons in the two face patches. Subjects were presented with fifteen dynamic natural movie clips of three unfamiliar monkeys issuing five different call varieties of differing emotional valence. The call types included eight affiliative coos, two agonistic tonal screams, two aggressive pant-threats, two barks, and one bark-growl (Figure 1C).[31–34] The acoustic structure ranged broadly, with coos and agonistic calls having more tonal elements and barks, pant-threats, and bark-growls having a broadband and atonal structure.[34] Trial sequences consisted of randomly interleaved presentations of each original audiovisual movie, the visual component only (i.e., silent movie), and auditory component only.

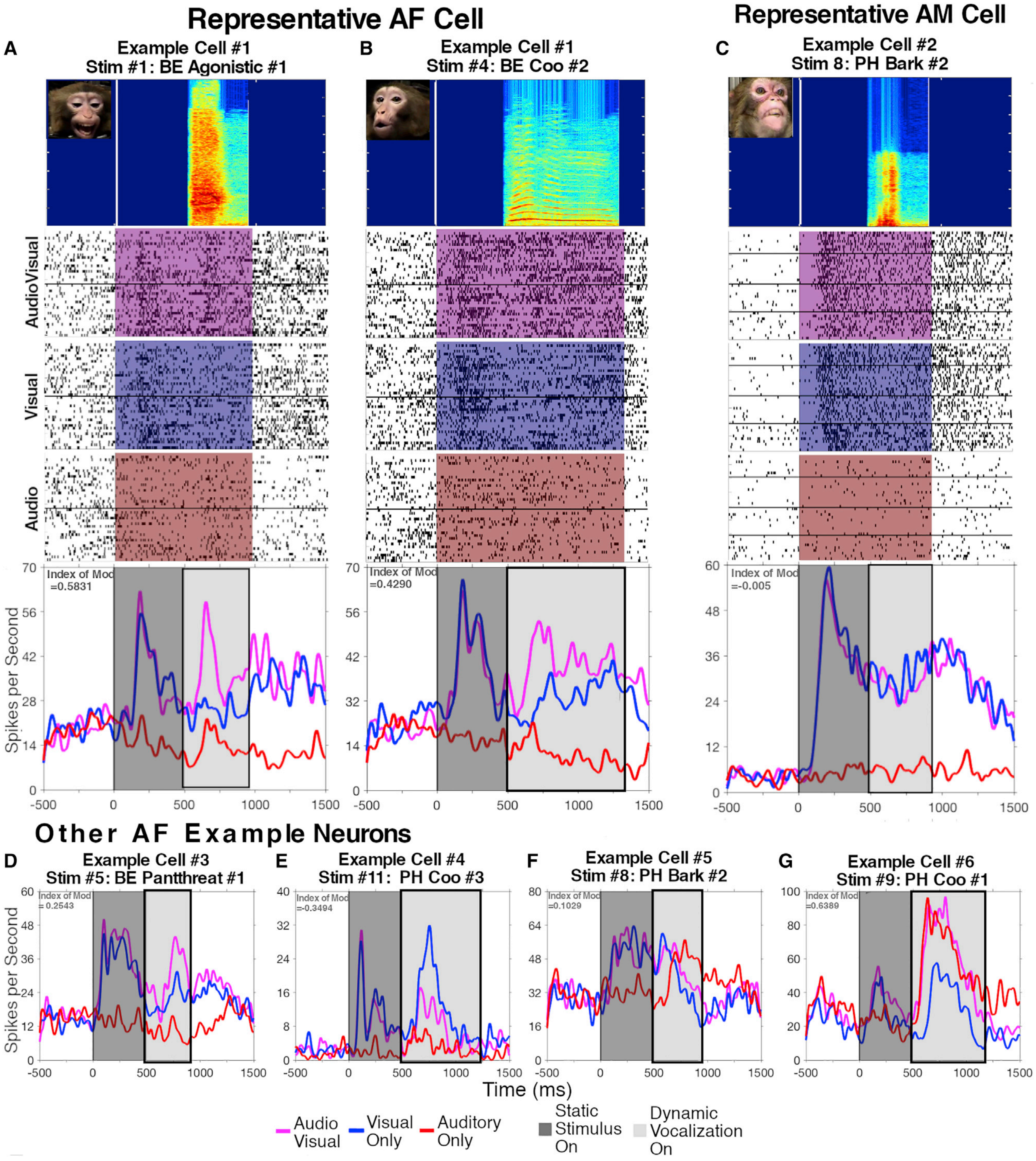

Figure 2. Example responses from AF and AM

The dark gray panel indicates the static frame although the light gray indicates the audiovisual movie stimulus (magenta), silent movie (blue), or vocalization (red).

(A and B) Typical enhancement of AF neuron's response for two different stimuli (two-way ANOVA; A, p Vis < 0.0001, p Aud = 0.3401, p Int < 0.0001; B, p Vis < 0.0001, p Aud = 0.8686, p Int < 0.0001). The horizontal black line within the rasters delineates the different recording sessions for the presented neurons.

(C) Typical AM neuron's response with little or no auditory modulation (p Vis < 0.0001; p Aud = 0.8014; p Int = 0.2002).

(D–G) Additional example AF neuron responses.

(D) Another typical AF non-linear multisensory enhanced response (p Vis < 0.0001; p Aud = 0.7110; p Int < 0.001).

(E–G) Different profiles of audiovisual integration also expressed by neurons in AF.

(E) A cell with a non-linear suppression of spiking in response to the audiovisual condition compared to the visual-only condition (p Vis < 0.0001; p Aud = 0.0086; p Int = 0.001).

(legend continued on next page)

AF face patch neurons

The majority of neurons in AF exhibited a significant auditory modulation of their visual responses in response to one or more of the vocalization stimuli. In addition, some AF neurons responded to the auditory component alone. The influence of acoustic information on AF responses took multiple different forms, which we qualitatively separated based on the characteristics of their response. The most commonly observed pattern was multisensory enhancement of the visual response (Figures 2A, 2B, and 2D). For neurons in this category, the auditory stimulus alone did not elicit a significant response but did elevate the neurons' response to the visual movie. The prominence of this pattern across the population was evident in the auditory enhancement observed in the grand average activity across all AF cells and all stimuli (Figure S1A). A smaller number of neurons exhibited multisensory suppression (Figure 2E), where the auditory stimulus diminished the neurons' visual response. Finally, a relatively small subset of neurons did respond to one or more auditory stimuli alone (Figures 2F and 2G). These neurons were generally bimodal, meaning that they responded to both the auditory stimuli and the visual stimuli. For such neurons, the magnitude of their response to an audiovisual stimulus typically matched that to the visual stimulus alone, though a few matched that of the auditory stimulus alone.

To quantitively evaluate auditory responses and audiovisual interactions, we determined the average spike rate during the dynamic period of the movie, beginning at 500 ms after the static frame presentation and ending 100 ms after the termination of the movie clip, which varied between stimuli. We conducted a two-way analysis of variance (ANOVA) on the spike rates for each movie separately or collapsed across all calls to determine the auditory or visual contributions, along with their interaction. Neurons were classified as visual if they showed a significant main effect only of the visual stimulus, auditory if they showed a significant main effect only of the auditory stimulus, linear multisensory if they showed a significant main effect for both the auditory and the visual stimulus, and non-linear multisensory if they showed a significant interaction term.

Based on this analysis across all calls, 76.0% of the 119 neurons recorded from the AF face patch were multisensory and exhibited a significant influence of the auditory component of the vocalization (two-way ANOVA $p < 0.01$; Figure 3B, top bar). Most prominently, 57.7% of neurons were classified as non-linear multisensory, most often showing a significant modulation of the visual response by the auditory component. Another 14.4% of neurons were classified as linear multisensory, as they exhibited a significant response to both auditory and visual stimuli presented alone, together with a roughly additive effect during the audiovisual condition. Finally, 3.8% of the neurons responded *only* to the auditory stimulus. For each individual vocalization movie, auditory modulation was observed in a subset (24.2%–50.6%) of neurons (Figure 3B, bars 1–15).

Multisensory cells showed considerable variation in the proportion of the 15 movie stimuli that elicited a response. Approximately one-third of neurons (31/102; 30.5%) exhibited auditory modulation to only a single stimulus, whereas another one-third of neurons (33/102; 32.5%) showed modulation to five or more stimuli (Figure 3G). To quantify the auditory effect on the visual responses, we calculated an index of auditory modulation (see STAR methods), collapsing values across all fifteen stimuli for each neuron (Figures 3E and 3F). The index values range from −1 to 1, where a negative index indicates an auditory suppression of the visual response and a positive index indicates an enhancement. The distribution of collapsed audiovisual index values for AF neurons centered around a median of 0.12, indicating a predominately enhanced spike rate modulation ($t_{(118)} = 5.317$; $p = 5 \times 10^{-7}$). The index of modulation revealed no strong preference for any particular stimulus across the population, although there were some differences between stimuli on average (Figure S2B). Further, the magnitude of acoustic modulation was not systematically related to visual responsiveness of the neuron (Figure 3E) and showed a non-significant relationship with the face selectivity index (Spearman correlation $\rho = 0.1110$; $p = 0.2296$; Figure S3). Together, these analyses demonstrate a prominent auditory modulation of visual responses to macaque vocalizations among AF face patch neurons, with the net effect being enhancement of selective visual responses across the population.

AM face patch neurons

We performed the same analyses for neurons recorded from the AM face patch, which is known to receive direct input from AF as well as from other multisensory regions.[26] In stark contrast to AF, few AM neurons were affected by the auditory component of the vocalization movies, and the grand average across all AM cells and stimuli showed little if any audiovisual modulation (Figures 2C and S1B). This contrast was most clearly reflected in the ANOVA analysis across all stimuli of AM neurons, revealing no significant auditory modulation of any neuron (Figure 3D). The audiovisual index across the population had a median of −0.02, which was not significantly different from zero ($t_{(54)} = -0.6271$; $p = 0.5332$; Figures 3E and 3F). For individual movies, only a few AM neurons ($n = 14$) showed significant auditory modulation. Of these neurons, 11/14 showed such modulation to a single stimulus, with the remaining 3 cells showing significant modulation to two stimuli (Figure 3G). These results indicate auditory modulation in area AM is rare and, when present, highly selective for particular stimuli or very weak. The difference in auditory contribution to the AF and AM face patches was underscored by the results of a linear mixed-effects model that included the face patch and modality as its variables (Table 1).

In summary, the results indicate that two high-level anterior face patches, AF and AM, differ sharply in their modulation by the auditory component of macaque vocalizations. The auditory influence in AF was conspicuous, widespread, and often extended to multiple stimuli, whereas that in AM was virtually nonexistent in our recordings. We next focused on the observed audiovisual modulation in face patch AF and, in particular, the requisite auditory and visual components of our stimuli.

(F and G) Bimodal responses, where the response to the audiovisual movie mirrored the response to a unimodal condition (visual in F, p Vis = 0.0396, p Aud < 0.0001, p Int = 0.05, and auditory in G, p Vis = 0.7750, p Aud < 0.0001, p Int = 0.2888) along with a response to the other unimodal stimulus. Response types were determined by two-way ANOVA considering the presence or absence of the audio and visual stimulus components and their interaction. The index of modulation is shown in the corner of each spike density function.
See also Figure S1.

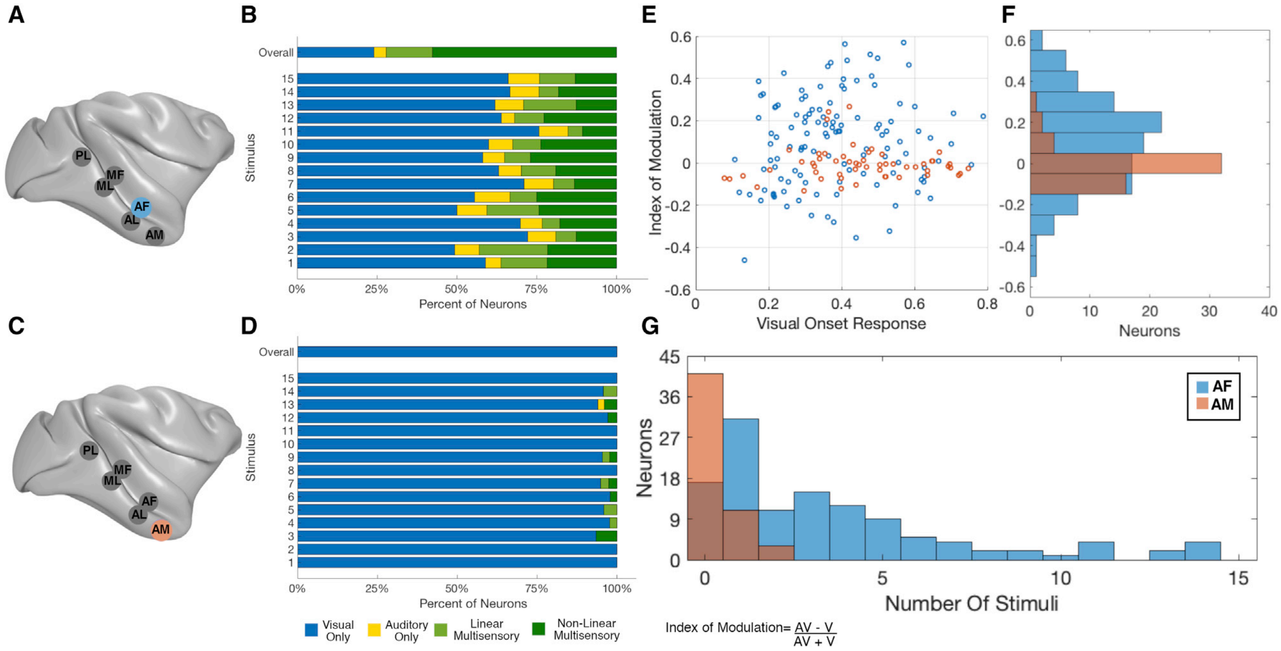

Figure 3. Comparison of population responses to audiovisual stimuli of AF and AM neurons
(A and C) Schematic representations of the relative positions of all the face patches specifically marking AF (A) and AM (C).
(B and D) Plot of the proportions of neurons with significant modulation to each modality or the combination of modalities as calculated by two-way ANOVA for all stimuli (top row) and each stimulus analyzed independently (lower rows; AF, n = 119; AM, n = 55).
(E) A scatterplot comparing the initial response the appearance of the still frame to the index of modulation for both AF and AM neurons.
(F) Distribution of the mean index of modulation for each neuron for AF (blue) and AM (orange); the black line marks 0, the dashed blue line indicates the median of the AF distribution (0.1290), and the dashed red line indicates the median for the AM distribution (−0.0150).
(G) Distribution of neurons for which a given number of stimuli demonstrate auditory modulation.
See also Figures S2 and S3.

Experiment 2: investigation of multisensory responses using macaque avatar

To examine audiovisual processing in the AF face patch further, we used a realistic macaque avatar stimulus,[35] whose facial movements were programmed to mimic real facial actions during the specific vocalizations. The macaque avatar allowed for the investigation of particular aspects of audiovisual integration while maintaining the same face identity, head angle, and other visual stimulus properties. For experiment 2, the avatar was animated to match five different calls (coo, agonistic, pant-threat, bark, and bark-growl) based on the original macaque movie clips (see STAR methods). Now, with this more-controlled visual component of the stimulus, we investigated two questions related to the specific features important to the observed audiovisual responses of AF neurons.

Critical role of the face

We first asked whether the observed auditory modulation would differ if the visual stimulus were a face, now in avatar form, versus a surrogate non-face stimulus. Specifically, we compared responses elicited by a vocalizing avatar (Figure 4A) to those found when the face was replaced by expanding and contracting dynamic disk, whose movements were matched to the changing mouth size and synchronized with the auditory track (Figure 4B).

Neural responses to the avatar, including the modulation by the corresponding auditory stimulus, were broadly similar to the original movies, albeit with a smaller fraction of neurons demonstrating multisensory responses (Figure 4C). We recorded 121 AF neurons in this experiment, an independent population from those recorded in experiment 1, and again used a two-way ANOVA to establish significant responses to each sensory modality. Of these neurons, 99/121 (81.8%) responded to at least one of the visual or auditory stimuli, although the remaining 22 were unresponsive to the experimental stimuli and excluded from further analysis. 26/99 (26.3%) neurons exhibited a significant response to the auditory component or significant auditory modulation to at least one of the five vocalization-movie call types (Figure 4C). The reduced proportion compared to the original faces likely reflects the imposition of a single avatar facial identity, as well as the lower overall number of stimuli. Importantly, very few neurons showed significant auditory modulation when the dynamic face was replaced with the dynamic disk (Figure 4B). Of the 99 neurons that responded to the avatar movie stimuli, only 5/99 (5.1%) neurons showed any linear or non-linear multisensory interaction with the disk movie control (Figure 4D). These responses suggest that the observed auditory modulation does not reflect a general temporal synchronization with visual movement but instead depends upon viewing facial structure.

Critical acoustic parameters

We next used the same avatar stimulus to investigate the relative importance of spectral versus temporal acoustic parameters in the modulation of visual responses. To this end, we repeated the experiment by pairing the avatar stimuli with temporally

CellPress
OPEN ACCESS

Table 1. Display of the results of linear mixed-effects model, evaluating the effect of each factor on the average spike rate

Factor	Beta coefficient (a.u.)	T-stat	Degrees of freedom	p value
Presence of auditory stim.	0.0186	1.9288	518	p = 0.0543
Presence of visual stim.	0.1321	14.4616	518	p < 0.0001
Interaction of face patch and auditory stim.	0.0339	3.87636	518	p = 0.0001
Face patch	−0.0674	−3.8036	518	p = 0.0002

The model shows a significant effect for the interaction of face patch and auditory stimuli, with the positive beta indicating that AF neurons respond more strongly than AM neurons to the addition of auditory stimulus. See also Figure S3.

patterned broadband noise (BBN) by applying the temporal envelopes of the original calls to carrier noise (1–20,000 Hz frequency range), such that the temporal structure was preserved, but the spectral content was disrupted.

Most AF neurons responded similarly to both normal audiovisual avatar stimuli and the matching audiovisual noise avatar stimuli (example shown in Figures 5A and 5B). Cells still responded to or were modulated by matched auditory noise despite the lack of detailed spectral information and did not differ significantly from the response to the normal vocalization (Figure 5C). A similar percentage (25/99; 25.3%) of neurons showed linear or non-linear modulation to the matched noise, and 5.1% responded to the noise stimulus alone. To directly compare these different auditory conditions, we conducted an ANOVA with the natural vocalization and matched noise as a factor and performed a post hoc pairwise comparison with a Tukey-Kramer test. Only 7/121 (5.8%) of neurons exhibited a significant difference between the matching noise and the natural vocalization.

To ensure this similarity was not driven solely by sensitivity to broadband vocalizations, we compared responses to the tonal coo and agonistic calls with their corresponding broadband controls. Even in cases of harmonic calls, the neurons continued to show similar responses between the audiovisual conditions and the matched BBN conditions (example in Figures 5D and 5E), at similar levels across the population (Figure 5F). Similar proportions of neurons exhibited multisensory modulation to both the harmonic calls (29.4%) and their matched noise controls (35.4%). These results suggest that, despite sensitivity to fine visual features, multisensory modulation in AF face patch is principally determined not by the fine spectral details of a vocalization but by its temporal structure.

DISCUSSION

Audiovisual modulation in face patches

Our results indicate that most AF face patch neurons are affected by concomitant auditory stimulation during the viewing of macaque vocalizations. Although previous research indicates that the anterior STS is a multisensory region[3,4,13,14] that contains cells with selective audiovisual responses to faces,[36–38] our data demonstrate, for the first time, this pattern is observed within a visually defined face patch. Though the predominant responses of the recorded AF cells were visual, most showed some level of auditory modulation to movies within our limited stimulus set, and some cells responded to one or more auditory vocalizations in the absence of any visual stimulus.

Previous explorations of the STS organization in monkeys and humans have indicated a patchy spatial organization across primate species, with unisensory regions for each modality and audiovisual regions clustering together.[4,37] In this context, the AF face patch might have been a good candidate for a visual-only region. However, our results instead suggest that this face patch may participate in audiovisual integration. The diverse expression of multisensory responses was striking. For example, some neurons responded to both auditory and visual stimuli alone but, when presented with the combined audiovisual stimulus, responded as if only one or the other unimodal stimulus had been presented. Other cells responded to static faces and then responded only to the vocalization presented, but not the moving face. Some of these results might be due to a high selectivity for individual identities, expressions, or other parameters of the movie, suggesting that estimates of multisensory responses would be greater with larger testing sets.[39]

The near absence of auditory modulation observed among AM neurons suggests that audiovisual modulation is expressed differentially among face patches. The contrast between AF and AM is particularly striking given that AM receives direct anatomical projections from AF and responds when AF receives electrical microstimulation.[25,26] It bears mention, however, that the focal nature of our electrophysiological sampling means that our sampling of AM was limited and that we therefore cannot rule out a stronger multisensory component in other portions of AM that were missed in the two monkeys tested. It is also possible that AM neurons may be responsive to other sensory stimuli through the inputs they receive from neighboring perirhinal and parahippocampal areas, which are known to carry somatosensory information.[28] In contrast to AM, the connections of the AF patch have not been directly assessed with retrograde tracers, so its specific connections are unknown. In general, the STS fundus receives input from multisensory areas, such as intraparietal and prefrontal regions, as well as unisensory association areas, including high-level auditory belt and parabelt cortex as well as visual inferior temporal TE and TEO cortex, both directly and indirectly through lateral regions of the STS.[7,9,11,39–43] Our results, combined with previous anatomical and electrophysiological finding, thus suggest that the AF face patch participates in multisensory integration that is typical for neighboring areas of the STS fundus.

Whether neurons in other face patches integrate auditory information in a manner similar to AF remains to be seen. The specific pattern of interconnections among face patches, and their arrangement into one or more hierarchies, is presently a matter of inquiry.[44] Auditory sensitivity adds a new property to the response selectivity of AF neurons, whose response profiles and covariation with other brain areas is already quite varied.[45] Based on the known layout of the temporal cortex and its relationship to audiovisual responses, one might guess that other face patches lying in the fundus (middle fundus [MF]) or upper

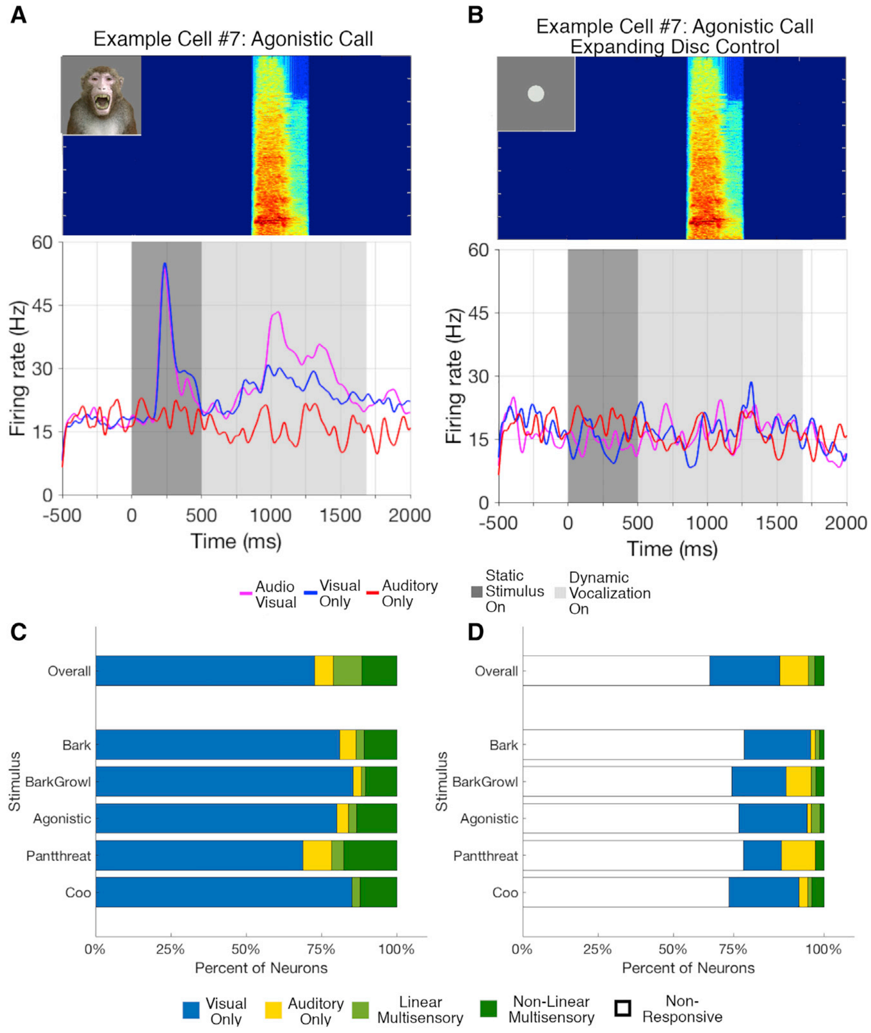

Figure 4. Responses to visual control stimuli

(A and B) Single-cell example of responses to the different versions of the agonistic call stimulus. (A) portrays the average responses to the avatar producing an agonistic call although (B) shows the response of the same cell to the expanding disk stimulus matched to the same vocalization.

(C and D) Population response of AF to audiovisual avatar stimuli comparing (C) the selectivity of cell responses to the audiovisual avatar stimuli to (D) the selectivity of cell responses to the audiovisual expanding disk control stimuli.

bank (middle dorsal [MD]] and anterior dorsal [AD]) of the STS might be good candidates for audiovisual integration. Notably, these face patches, like AF, generally exhibit a sensitivity to facial motion,[21] which may be central to the synchronization of visual and auditory information during a vocalization. By contrast, area AM on the ventral surface of the temporal lobe is more commonly associated with processing of individual facial identities.[27] It, like the recently described perirhinal (PR) and temporal pole (TP) areas involved in face familiarity,[46] may be less governed by dynamic facial behaviors and more by facial features. Frontal lobe face patches, prefrontal orbital (PO), prefrontal arcuate (PA), and prefrontal lateral (PL), are known to respond to expressive faces similar to fundus patches[47,48] and are coextensive with prefrontal cortical areas that have been shown to be responsive to vocal stimuli and to their combination with facial gestures, including many of the same stimuli used in the present study.[39,49] These patches may also integrate audiovisual signals in a way that is yet to be elucidated. Further study of these functionally defined regions is needed, including investigation of their specific anatomical interconnections, their participation in multisensory integration, and their roles in reciprocal social communication.

Audiovisual selectivity

Given AF neurons' selectivity for particular movies, the macaque avatar allowed for a controlled examination of key variables. The virtual absence of auditory modulation for the temporally synchronized dynamic disk stimulus is consistent with the assumed specialization for faces within the face patch network. These responses indicate that AF neurons specifically combine auditory stimuli with facial information, rather than any temporally synchronized visual object. In previous studies, the temporal congruence between a visual stimulus and its auditory pair has been an important feature in multisensory integration in the STS although call type or spectral detail has shown little effect.[36,50] Indeed, we found that the temporal structure alone, even when applied to broadband noise, was sufficient to elicit auditory modulation of visual responses to a face. Thus, the relative unimportance of auditory spectral content compared to visual input may thus be a characteristic of multisensory integration in the fundus of the STS. Interestingly, nearly the converse was observed in an fMRI-defined voice-specific area, a high-level auditory area on the supratemporal plane of the macaque temporal lobe. In that area, audiovisual neurons expressed selectivity for acoustic vocalizations although visual modulation of the acoustic response exhibited little selectivity to the visual stimulus.[36,51,52] Together, these results suggest an overall principle of multisensory integration in high-level sensory areas, wherein highly stimulus-selective responses for the primary modality can be modulated by a relatively broad range of temporally synchronized stimuli presented in the secondary modality.

Broader implications

The face-dependent multisensory responses of AF neurons to vocalizations indicate that this area may participate in a larger cortical network in the service of social communication. For example, regions of the macaque auditory cortex also exhibit multisensory modulation tied to visual

CellPress
OPEN ACCESS

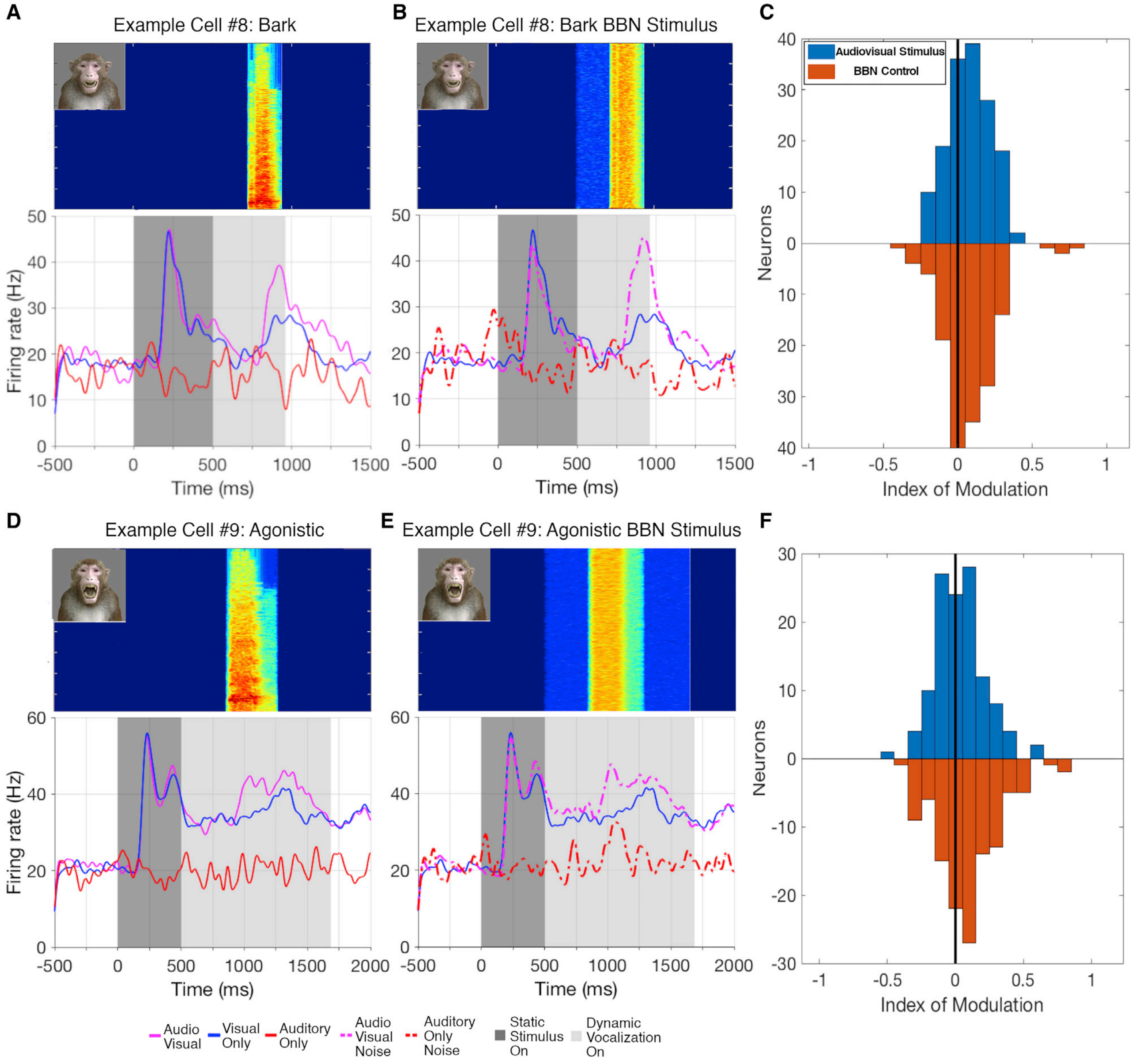

Figure 5. Responses to acoustic control stimuli

(A and B) A single-cell example of responses to the different versions of the bark stimulus with (A) the average response of a single cell to the avatar bark stimulus and (B) the response to the avatar when a temporally modulated broadband noise (BBN) stimulus replaced the bark vocalization.

(C) The distribution of the index of modulation for all calls across the population for both the avatar audiovisual stimuli and the avatar BBN control stimuli.

(D and E) A single-cell example of the response to different versions of the agonistic call with (D) the cell response to the avatar agonistic stimulus and (E) the response to the avatar agonistic BBN stimulus.

(F) The distribution of index of modulation to the tonal coos and agonistic calls.

presentation of faces and face information, with the visual response component likely arising from well-known reciprocal connections with the STS.[7,11,34,53–55] Our results suggest that, within the STS, the AF face patch may be an important region involved in this processing. Projections between these areas may reflect a conserved multisensory pathway. Anatomical and physiological interaction between face-voice areas in humans are thought to mediate vocal communication.[56,57] The STS also feeds forward to and receives feedback projections from the VLPFC, which also contains high proportions of face-selective audiovisual neurons[39,49,58] and is itself thought to draw upon visual, auditory, and multisensory areas.[12,59] Further studies are required to establish the specific anatomical and functional connections of AF with other multimodal, affective, and voice-selective regions and, more importantly, what role it plays within the larger range of multisensory areas. At present, the results draw attention to a well-known face-

selective area, whose integration of vocal auditory signals into its visual analysis makes it a likely contributor to primates' advanced skills in the domain of multisensory social perception.

STAR★METHODS

Detailed methods are provided in the online version of this paper and include the following:

- KEY RESOURCES TABLE
- RESOURCE AVAILABILITY
  - Lead contact
  - Materials availability
  - Data and code availability
- EXPERIMENTAL MODEL AND SUBJECT DETAILS
  - Subjects
- METHOD DETAILS
  - fMRI
  - Experiment design
  - Stimuli
  - Electrophysiology recording
- QUANTIFICATION AND STATISTICAL ANALYSIS
  - Longitudinal recording
  - Data analysis

SUPPLEMENTAL INFORMATION

Supplemental Information can be found online at https://doi.org/10.1016/j.cub.2021.01.102.

ACKNOWLEDGMENTS

The authors would like to thank Kenji Koyano, Elena Waidmann, Katy Smith, David Yu, Frank Ye, and Charles Zhu for their assistance with this work. Thanks to Julien Duchemin for his professional 3D modeling and rigging. This work also utilized the resources of the NIH High Performance Computing Core (https://hpc.nih.gov/). This research was supported by the Intramural Research Program of the National Institute of Mental Health (ZIA MH002898), the Wellcome Trust/Royal Society Fellowship (098418/Z/12/Z), and the National Institute of Deafness and Other Communication Disorders (R01DC04845).

AUTHOR CONTRIBUTIONS

A.P.K., A.P.M., and L.M.R. created the stimuli. A.P.K. conducted the experiments. A.P.K. analyzed the results. A.P.K., L.M.R., J.K.B., and D.A.L. interpreted the results. A.P.K., J.K.B., and D.A.L. wrote the manuscript.

DECLARATION OF INTERESTS

The authors declare no competing interests.

Received: January 18, 2020
Revised: December 29, 2020
Accepted: January 28, 2021
Published: February 25, 2021

REFERENCES

1. Ghazanfar, A.A., and Santos, L.R. (2004). Primate brains in the wild: the sensory bases for social interactions. Nat. Rev. Neurosci. *5*, 603–616.
2. Barraclough, N.E., and Perrett, D.I. (2011). From single cells to social perception. Philos. Trans. R. Soc. Lond. B Biol. Sci. *366*, 1739–1752.
3. Beauchamp, M.S., Lee, K.E., Argall, B.D., and Martin, A. (2004). Integration of auditory and visual information about objects in superior temporal sulcus. Neuron *41*, 809–823.
4. Beauchamp, M.S., Argall, B.D., Bodurka, J., Duyn, J.H., and Martin, A. (2004). Unraveling multisensory integration: patchy organization within human STS multisensory cortex. Nat. Neurosci. *7*, 1190–1192.
5. Barraclough, N.E., Xiao, D., Baker, C.I., Oram, M.W., and Perrett, D.I. (2005). Integration of visual and auditory information by superior temporal sulcus neurons responsive to the sight of actions. J. Cogn. Neurosci. *17*, 377–391.
6. Ghazanfar, A.A., and Schroeder, C.E. (2006). Is neocortex essentially multisensory? Trends Cogn. Sci. *10*, 278–285.
7. Seltzer, B., and Pandya, D.N. (1978). Afferent cortical connections and architectonics of the superior temporal sulcus and surrounding cortex in the rhesus monkey. Brain Res. *149*, 1–24.
8. Pandya, D.N., and Seltzer, B. (1982). Association areas of the cerebral cortex. Trends Neurosci. *5*, 386–390.
9. Hackett, T.A., Stepniewska, I., and Kaas, J.H. (1998). Subdivisions of auditory cortex and ipsilateral cortical connections of the parabelt auditory cortex in macaque monkeys. J. Comp. Neurol. *394*, 475–495.
10. Kaas, J.H., and Hackett, T.A. (2000). Subdivisions of auditory cortex and processing streams in primates. Proc. Natl. Acad. Sci. USA *97*, 11793–11799.
11. Seltzer, B., and Pandya, D.N. (1994). Parietal, temporal, and occipital projections to cortex of the superior temporal sulcus in the rhesus monkey: a retrograde tracer study. J. Comp. Neurol. *343*, 445–463.
12. Romanski, L.M., Bates, J.F., and Goldman-Rakic, P.S. (1999). Auditory belt and parabelt projections to the prefrontal cortex in the rhesus monkey. J. Comp. Neurol. *403*, 141–157.
13. Benevento, L.A., Fallon, J., Davis, B.J., and Rezak, M. (1977). Auditory–visual interaction in single cells in the cortex of the superior temporal sulcus and the orbital frontal cortex of the macaque monkey. Exp. Neurol. *57*, 849–872.
14. Bruce, C., Desimone, R., and Gross, C.G. (1981). Visual properties of neurons in a polysensory area in superior temporal sulcus of the macaque. J. Neurophysiol. *46*, 369–384.
15. Baylis, G.C., Rolls, E.T., and Leonard, C.M. (1987). Functional subdivisions of the temporal lobe neocortex. J. Neurosci. *7*, 330–342.
16. Hikosaka, K., Iwai, E., Saito, H., and Tanaka, K. (1988). Polysensory properties of neurons in the anterior bank of the caudal superior temporal sulcus of the macaque monkey. J. Neurophysiol. *60*, 1615–1637.
17. Kanwisher, N., McDermott, J., and Chun, M.M. (1997). The fusiform face area: a module in human extrastriate cortex specialized for face perception. J. Neurosci. *17*, 4302–4311.
18. Tsao, D.Y., Freiwald, W.A., Knutsen, T.A., Mandeville, J.B., and Tootell, R.B. (2003). Faces and objects in macaque cerebral cortex. Nat. Neurosci. *6*, 989–995.
19. Tsao, D.Y., Moeller, S., and Freiwald, W.A. (2008). Comparing face patch systems in macaques and humans. Proc. Natl. Acad. Sci. USA *105*, 19514–19519.
20. Perrett, D.I., Hietanen, J.K., Oram, M.W., and Benson, P.J. (1992). Organization and functions of cells responsive to faces in the temporal cortex. Philos. Trans. R. Soc. Lond. B Biol. Sci. *335*, 23–30.
21. Fisher, C., and Freiwald, W.A. (2015). Contrasting specializations for facial motion within the macaque face-processing system. Curr. Biol. *25*, 261–266.
22. Tsao, D.Y., Freiwald, W.A., Tootell, R.B., and Livingstone, M.S. (2006). A cortical region consisting entirely of face-selective cells. Science *311*, 670–674.
23. Bell, A.H., Malecek, N.J., Morin, E.L., Hadj-Bouziane, F., Tootell, R.B., and Ungerleider, L.G. (2011). Relationship between functional magnetic resonance imaging-identified regions and neuronal category selectivity. J. Neurosci. *31*, 12229–12240.

24. Aparicio, P.L., Issa, E.B., and DiCarlo, J.J. (2016). Neurophysiological organization of the middle face patch in macaque inferior temporal cortex. J. Neurosci. *36*, 12729–12745.

25. Moeller, S., Freiwald, W.A., and Tsao, D.Y. (2008). Patches with links: a unified system for processing faces in the macaque temporal lobe. Science *320*, 1355–1359.

26. Grimaldi, P., Saleem, K.S., and Tsao, D. (2016). Anatomical connections of the functionally defined "face patches" in the macaque monkey. Neuron *90*, 1325–1342.

27. Freiwald, W.A., and Tsao, D.Y. (2010). Functional compartmentalization and viewpoint generalization within the macaque face-processing system. Science *330*, 845–851.

28. Miyashita, Y. (2019). Perirhinal circuits for memory processing. Nat. Rev. Neurosci. *20*, 577–592.

29. Bondar, I.V., Leopold, D.A., Richmond, B.J., Victor, J.D., and Logothetis, N.K. (2009). Long-term stability of visual pattern selective responses of monkey temporal lobe neurons. PLoS ONE *4*, e8222.

30. McMahon, D.B., Jones, A.P., Bondar, I.V., and Leopold, D.A. (2014). Face-selective neurons maintain consistent visual responses across months. Proc. Natl. Acad. Sci. USA *111*, 8251–8256.

31. Gouzoules, S., Gouzoules, H., and Marler, P. (1984). Rhesus monkey (*Macaca mulatta*) screams: representational signalling in the recruitment of agonistic aid. Anim. Behav. *32*, 182–193.

32. Hauser, M.D. (1991). Sources of acoustic variation in rhesus macaque (*Macaca mulatta*) vocalizations. Ethology *89*, 29–46.

33. Hauser, M.D., and Marler, P. (1993). Food-associated calls in rhesus macaques (*Macaca mulatta*): I. Socioecological factors. Behav. Ecol. *4*, 194–205.

34. Romanski, L.M., Averbeck, B.B., and Diltz, M. (2005). Neural representation of vocalizations in the primate ventrolateral prefrontal cortex. J. Neurophysiol. *93*, 734–747.

35. Murphy, A.P., and Leopold, D.A. (2019). A parameterized digital 3D model of the Rhesus macaque face for investigating the visual processing of social cues. J. Neurosci. Methods *324*, 108309.

36. Perrodin, C., Kayser, C., Logothetis, N.K., and Petkov, C.I. (2014). Auditory and visual modulation of temporal lobe neurons in voice-sensitive and association cortices. J. Neurosci. *34*, 2524–2537.

37. Dahl, C.D., Logothetis, N.K., and Kayser, C. (2009). Spatial organization of multisensory responses in temporal association cortex. J. Neurosci. *29*, 11924–11932.

38. Ghazanfar, A.A., Chandrasekaran, C., and Logothetis, N.K. (2008). Interactions between the superior temporal sulcus and auditory cortex mediate dynamic face/voice integration in rhesus monkeys. J. Neurosci. *28*, 4457–4469.

39. Sugihara, T., Diltz, M.D., Averbeck, B.B., and Romanski, L.M. (2006). Integration of auditory and visual communication information in the primate ventrolateral prefrontal cortex. J. Neurosci. *26*, 11138–11147.

40. Seltzer, B., and Pandya, D.N. (1984). Further observations on parieto-temporal connections in the rhesus monkey. Exp. Brain Res. *55*, 301–312.

41. Seltzer, B., and Pandya, D.N. (1989). Frontal lobe connections of the superior temporal sulcus in the rhesus monkey. J. Comp. Neurol. *281*, 97–113.

42. Seltzer, B., and Pandya, D.N. (1989). Intrinsic connections and architectonics of the superior temporal sulcus in the rhesus monkey. J. Comp. Neurol. *290*, 451–471.

43. Cappe, C., Rouiller, E.M., and Barone, P. (2009). Multisensory anatomical pathways. Hear. Res. *258*, 28–36.

44. Freiwald, W.A. (2020). The neural mechanisms of face processing: cells, areas, networks, and models. Curr. Opin. Neurobiol. *60*, 184–191.

45. Park, S.H., Russ, B.E., McMahon, D.B.T., Koyano, K.W., Berman, R.A., and Leopold, D.A. (2017). Functional subpopulations of neurons in a macaque face patch revealed by single-unit fMRI mapping. Neuron *95*, 971–981.e5.

46. Landi, S.M., and Freiwald, W.A. (2017). Two areas for familiar face recognition in the primate brain. Science *357*, 591–595.

47. Tsao, D.Y., Schweers, N., Moeller, S., and Freiwald, W.A. (2008). Patches of face-selective cortex in the macaque frontal lobe. Nat. Neurosci. *11*, 877–879.

48. Taubert, J., Japee, S., Murphy, A.P., Tardiff, C.T., Koele, E.A., Kumar, S., Leopold, D.A., and Ungerleider, L.G. (2020). Parallel processing of facial expression and head orientation in the macaque brain. J. Neurosci. *40*, 8119–8131.

49. Diehl, M.M., and Romanski, L.M. (2014). Responses of prefrontal multisensory neurons to mismatching faces and vocalizations. J. Neurosci. *34*, 11233–11243.

50. Dahl, C.D., Logothetis, N.K., and Kayser, C. (2010). Modulation of visual responses in the superior temporal sulcus by audio-visual congruency. Front. Integr. Nueurosci. *4*, 10.

51. Petkov, C.I., Kayser, C., Steudel, T., Whittingstall, K., Augath, M., and Logothetis, N.K. (2008). A voice region in the monkey brain. Nat. Neurosci. *11*, 367–374.

52. Perrodin, C., Kayser, C., Logothetis, N.K., and Petkov, C.I. (2011). Voice cells in the primate temporal lobe. Curr. Biol. *21*, 1408–1415.

53. Ghazanfar, A.A., Maier, J.X., Hoffman, K.L., and Logothetis, N.K. (2005). Multisensory integration of dynamic faces and voices in rhesus monkey auditory cortex. J. Neurosci. *25*, 5004–5012.

54. Kayser, C., Petkov, C.I., Augath, M., and Logothetis, N.K. (2007). Functional imaging reveals visual modulation of specific fields in auditory cortex. J. Neurosci. *27*, 1824–1835.

55. Kayser, C., Logothetis, N.K., and Panzeri, S. (2010). Visual enhancement of the information representation in auditory cortex. Curr. Biol. *20*, 19–24.

56. von Kriegstein, K., Kleinschmidt, A., Sterzer, P., and Giraud, A.L. (2005). Interaction of face and voice areas during speaker recognition. J. Cogn. Neurosci. *17*, 367–376.

57. Blank, H., Anwander, A., and von Kriegstein, K. (2011). Direct structural connections between voice- and face-recognition areas. J. Neurosci. *31*, 12906–12915.

58. Romanski, L.M., and Hwang, J. (2012). Timing of audiovisual inputs to the prefrontal cortex and multisensory integration. Neuroscience *214*, 36–48.

59. Romanski, L.M., Tian, B., Fritz, J., Mishkin, M., Goldman-Rakic, P.S., and Rauschecker, J.P. (1999). Dual streams of auditory afferents target multiple domains in the primate prefrontal cortex. Nat. Neurosci. *2*, 1131–1136.

60. Cox, R.W. (1996). AFNI: software for analysis and visualization of functional magnetic resonance neuroimages. Comput. Biomed. Res. *29*, 162–173.

61. Eastman, K.M., and Huk, A.C. (2012). PLDAPS: a hardware architecture and software toolbox for neurophysiology requiring complex visual stimuli and online behavioral control. Front. Neuroinform. *6*, 1.

62. Quiroga, R.Q., Nadasdy, Z., and Ben-Shaul, Y. (2004). Unsupervised spike detection and sorting with wavelets and superparamagnetic clustering. Neural Comput. *16*, 1661–1687.

STAR★METHODS

KEY RESOURCES TABLE

REAGENT or RESOURCE	SOURCE	IDENTIFIER
Experimental models: organisms/strains		
Rhesus Macaque (Macacca Mulatta)	NIMH/NIH	N/A
Software and algorithms		
MATLAB	MathWorks	RRID: SCR_001622
AFNI	AFNI	RRID: SCR_005927
Psychophysics Toolbox	Psychtoolbox	RRID: SCR_002881
PLDAPS	HukLab	N/A
Blender	Blender Foundation	RRID: SCR_008606
Other		
DataPixx	VPixx Technologies	RRID: SCR_009648
EyeLink	SR Research	RRID: SCR_009602

RESOURCE AVAILABILITY

The raw data supporting this study is not available in a public repository because of complex custom data formats and the size of the files but are available from the lead contact upon request.

Lead contact

Further information and requests for reagent should be directed to lead contact David A. Leopold (leopoldd@mail.nih.gov)

Materials availability

This study did not generate any new materials or reagents.

Data and code availability

The raw data supporting this study is not available in a public repository because of complex custom data formats and the size of the files but are available from the lead contact upon request.

EXPERIMENTAL MODEL AND SUBJECT DETAILS

Subjects

Four rhesus macaque monkeys designated SP (Monkey 1, 9 kg), SR (Monkey 2, 10 kg), W (Monkey 3, 11kg), and M (Monkey 4, 9 kg), were implanted with a single chronic microwire bundles fixed within a custom MRI-compatible chambers and microdrive assembly. The electrode bundles were advanced post-surgically to achieve proper depth of recording. The electrodes in Monkey SP were located in the right hemisphere, whereas those in monkeys SR, M, W were in the left hemisphere. All procedures were approved by the Animal Care and Use Committee of the National Institute of Mental Health.

METHOD DETAILS

fMRI

Functional and anatomical magnetic resonance imaging (MRI) was conducted in the Neurophysiology Imaging Facility Core (NEI, NIMH, NINDS) using a vertical 4.7T Bruker Biospin scanner. For all subjects, hemodynamic responses were enhanced by injection with monocrystalline iron-oxide nanoparticles (MION). Details of scanning and stimulus presentation are described further in[30]. Briefly, Monkey SP underwent a standard block design localizer consisting of 24 s blocks of images of static macaque faces contrasted with blocks of images of non-face objects. In monkeys SR, M, and W, the blocks consisted of short movie clips of macaques making facial expressions contrasted with short movie clips of moving scenes and moving objects. Subjects received a juice reward for maintaining fixation every 2 s. All fMRI data was analyzed with AFNI[60] and custom software developed in MATLAB (Mathworks, Natwick, MA).

CellPress
OPEN ACCESS

Experiment design

All subjects performed a viewing task. The subject initiated the trial by fixating on a 0.7° crosshair within a window of 2° visual angle for between 200-300ms. A stimulus was then presented in either an audiovisual, visual only, or audio only format. For the audio only condition, the fixation marker remained on the screen and the subject was required to maintain fixation. For the audiovisual and visual conditions, a visual stimulus appeared in a square 10° visual angle window, and the subject was allowed to freely view any part of the stimulus. An infrared camera (Eyelink II, SR Research) monitored the subject's gaze as it performed this task and trials were aborted if the subject looked outside the window for longer than 100ms. All visual stimuli were presented on an OLED 4k Monitor 95cm from the subject using a graphical user interface (GUI) derived from PLDAPS (further described here[61]) in MATLAB. All auditory stimuli were presented in mono from two Tannoy Reveal Speakers placed on the edges of the monitor to create the percept the sound originated from the center of the screen. All auditory stimuli were projected at 65-80 dB SPL, verified using a Brüel and Kjaer (Denmark) sound level meter and at the full frequency range available.

Stimuli

In Experiment 1, stimuli consisted of short movies of monkeys vocalizing. The short movie clips featured macaque calls of varied acoustic structure and with a range of referential meaning and valence including affiliative coos, aggressive pant-threats, barks, and bark-growls, and agonistic/submissive screams[31–34]. Of these calls, the agonistic and coo calls used here had tonal/harmonic elements, while the remaining calls were broadband[34].These movies featured three individual monkeys at a variety of head positions (Figure 1C).

For Experiment 2, we selected five vocalization movie exemplars that represented each of the different call types described above. We then matched the mouth movements of the computer-generated animated macaque avatar[35] to the vocalization onset and envelope in each call to create new audiovisual movies (Figure 1D). The avatar enabled us to hold the basic visual appearance of a macaque face constant, including its identity and 3D head orientation, while its facial actions and mouth movements were animated and synchronized with the true macaque vocalizations. For this, we independently controlled features such as size of the mouth opening and amount of lip motion from the original movies using a GUI developed in MATLAB and animated the macaque avatar to follow the same patterns of motion. Movies of these avatar-vocalizations were rendered and compiled using the software Blender (the Blender Foundation). Frames were added to the avatar clips to extrapolate starting from or returning to a neutral facial expression before or after the vocalization.

In addition to the avatar stimuli, the monkey subject was presented with two categories of other control stimuli to determine the selectivity of audiovisual responses. Both sets of controls maintained the original temporal dynamics of the call structures. The first set controlled for visual motion. We replaced the macaque movie video with a dynamic disc whose instantaneous size was matched to the amplitude of the movements of the monkey's mouth in the original movie[39,53]. This control was designed to determine whether auditory modulation is face-specific or would be found with any temporally synchronized visual stimulus. In the second set of controls the audio track was manipulated by replacing the original spectral content with broadband noise convolved with the envelope of the original vocalization. This control evaluated the contribution of the spectral information on the acoustic modulation of neural responses.

To remove the contribution of known transient responses following the initial onset of a visual stimulus, all stimuli were introduced with a 500 ms static image prior to the onset of the movie movement and acoustic vocalization. Thus, the onset of the vocalization movie began after the face had already been on the display for 500 ms. This paradigm enabled us to deconvolve visual transient effects from the response to motion and addition of audio as well as approach a more naturalistic paradigm.

Electrophysiology recording

Following fMRI localization of the relevant face patches, subjects were implanted with 64 channel NiCr microwire bundle arrays fabricated by Microprobes for extracellular recording. Monkeys SR and SP received implants in face patch AF (an overlay of functional activation in Monkey SP is shown in Figure 1A). Monkeys M and W were implanted in face patch AM (functional overlay of Monkey W is shown in Figure 1B). All recordings were conducted in a radio shielded room (ETS-Lingreen) with a RZ2 BioAmp processor (Tucker-Davis Technologies) with a 128-channel capacity collecting a broadband signal of 0.5Hz-20KHz.

QUANTIFICATION AND STATISTICAL ANALYSIS

Longitudinal recording

One feature of the microwire arrays is the capacity for long-term longitudinal recordings of individual neurons, verified through similarity in waveform and selectivity fingerprints across days. All spike sorting was performed offline. Spikes were sorted using the wave_clus spike sorting package[62] and utilized the computational resources of the NIH HPC Biowulf cluster (https://hpc.nih.gov). To ensure cells were consistent across days, monkeys viewed a "fingerprinting" stimulus set consisting of 60 images containing sets of face categories (monkey faces and human faces) and non-face categories (objects and scenes). Face cells maintain selective responses across days[30]; therefore, by evaluating selectivity to a consistent stimulus set we can combine cell responses across days and months for the same cell. Responses were matched principally based on the pattern of selectivity to the "fingerprinting" stimulus set as well as the spike waveform and basic distribution of interspike intervals. Responses to these stimuli were also used to compute a face selectivity index (FSI) to quantify the strength of selectivity (Equation 1): FSI = (response_face – response_nonface)/ (response_face + response_nonface)

Data analysis

Following spike sorting and concatenation of individual cell responses across days, data were analyzed using custom software also created in MATLAB. We isolated a response window between 500ms (the end of the still frame and the beginning of motion) and 100ms after the conclusion of the movie stimulus and a baseline window between −300 and 0ms before the onset of the still frame. These windows were used for all further stimulus analysis. We conducted a two-way analysis of variance test (ANOVA) comparing response for the presence and absence of each component stimulus against the baseline response (further described in[39]) Neurons were classed as visual only or auditory only if they showed a significant response to either of the component stimuli alone, linear multisensory if they showed a significant main effect of both modalities, and non-linear multisensory if they had a significant interaction term. We also combined all stimuli into a single combined ANOVA again with each modality as a factor to assess the effect of each modality overall. Additionally, for the spectral controls, we conducted a pairwise comparison using a Tukey-Kramer test between the spectral controls, natural vocalization, and the silent condition to directly compare the effect of different auditory components.

For both Experiments 1 and 2, the main analysis compared the response to the audiovisual stimulus to the corresponding response to the visual stimuli alone. To this end, we calculated an index of modulation (Equation 2): Index of modulation = AV-V/AV+V. All rates were computed following baseline subtraction. Here, AV is the mean response to an audiovisual stimulus for a particular cell, whereas V is the mean baseline subtracted response to the visual only counterpart of that stimulus. This index enabled us to quantify the magnitude of modulation induced by auditory stimuli with an index between 1 and −1 with a positive index indicating that the addition of acoustic stimuli enhanced the response and a negative response indicating that acoustic stimuli suppressed the response.

Finally, we created a linear mixed-effect model to determine the effects of face patch independent of individual cell responses. Taking the average across all stimuli for each modality, we examined the effect of face patch, presence or absence of visual stimulus, and presence or absence of auditory stimulus on the average spiking rate. Each of these factors served a fixed-effect variable whereas the different cells were classed as a random effect. Through this model, we could isolate the exact effect of face patch and its interaction with stimulus type independent of variance between cells.

Current Biology, Volume *31*

Supplemental Information

Audiovisual integration

in macaque face patch neurons

Amit P. Khandhadia, Aidan P. Murphy, Lizabeth M. Romanski, Jennifer K. Bizley, and David A. Leopold

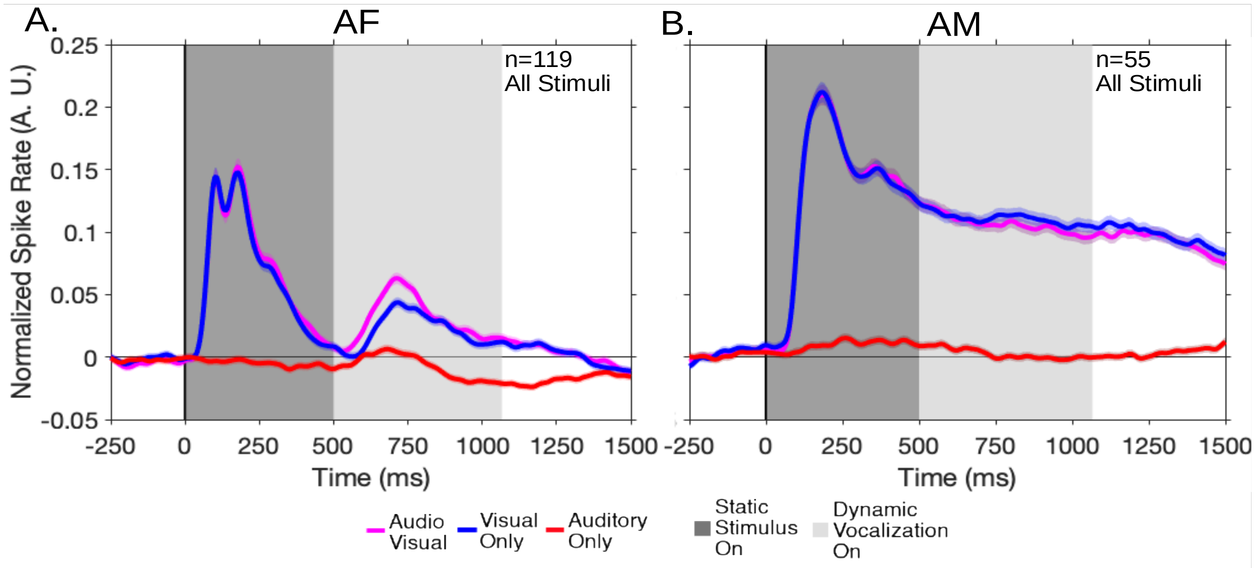

Figure S1. Grand average responses across all cells and all stimuli for face patches, related to Figure 2. A, B SDF plots of the grand average for AF, AM respectively (the SEM for each response is shown in a cloud surrounding each line with a very low SEM for AF)

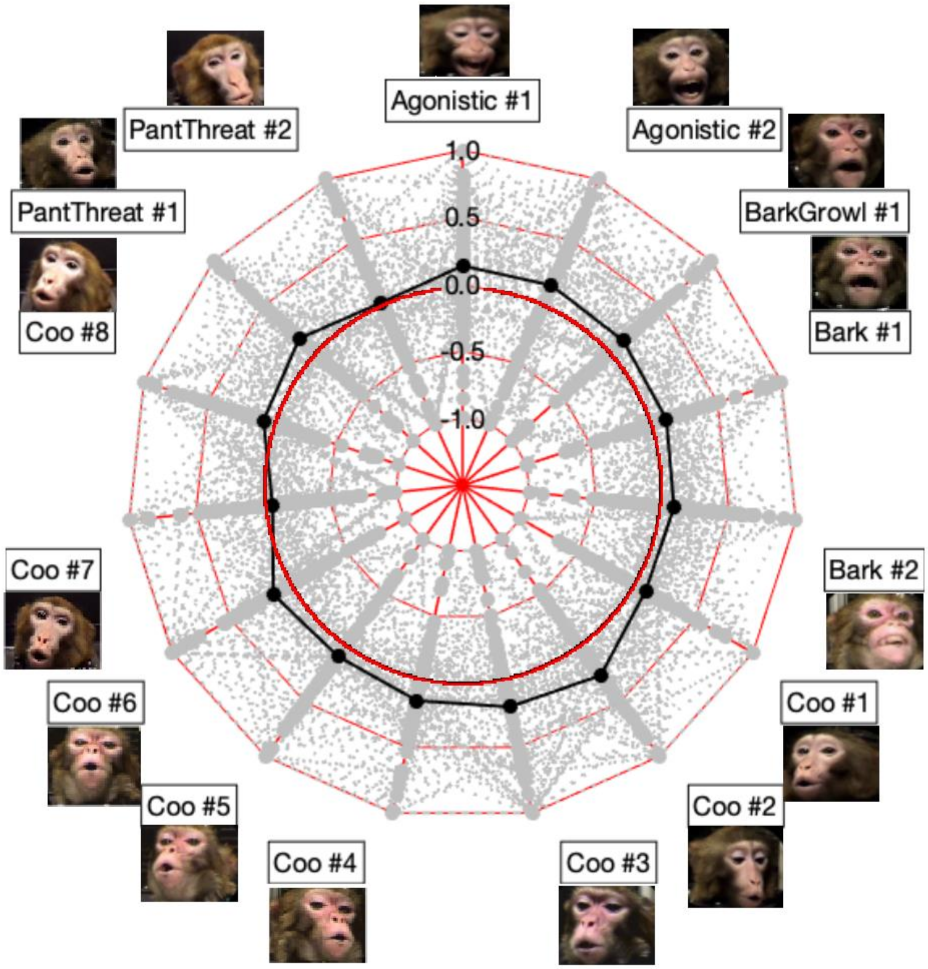

Figure S2 Effects of different stimuli on audiovisual integration, related to Figure 3. A radar plot showing the AV index for all cells in AF for each stimulus in the light gray with the mean represented in the black line and the zero mark in the red line.

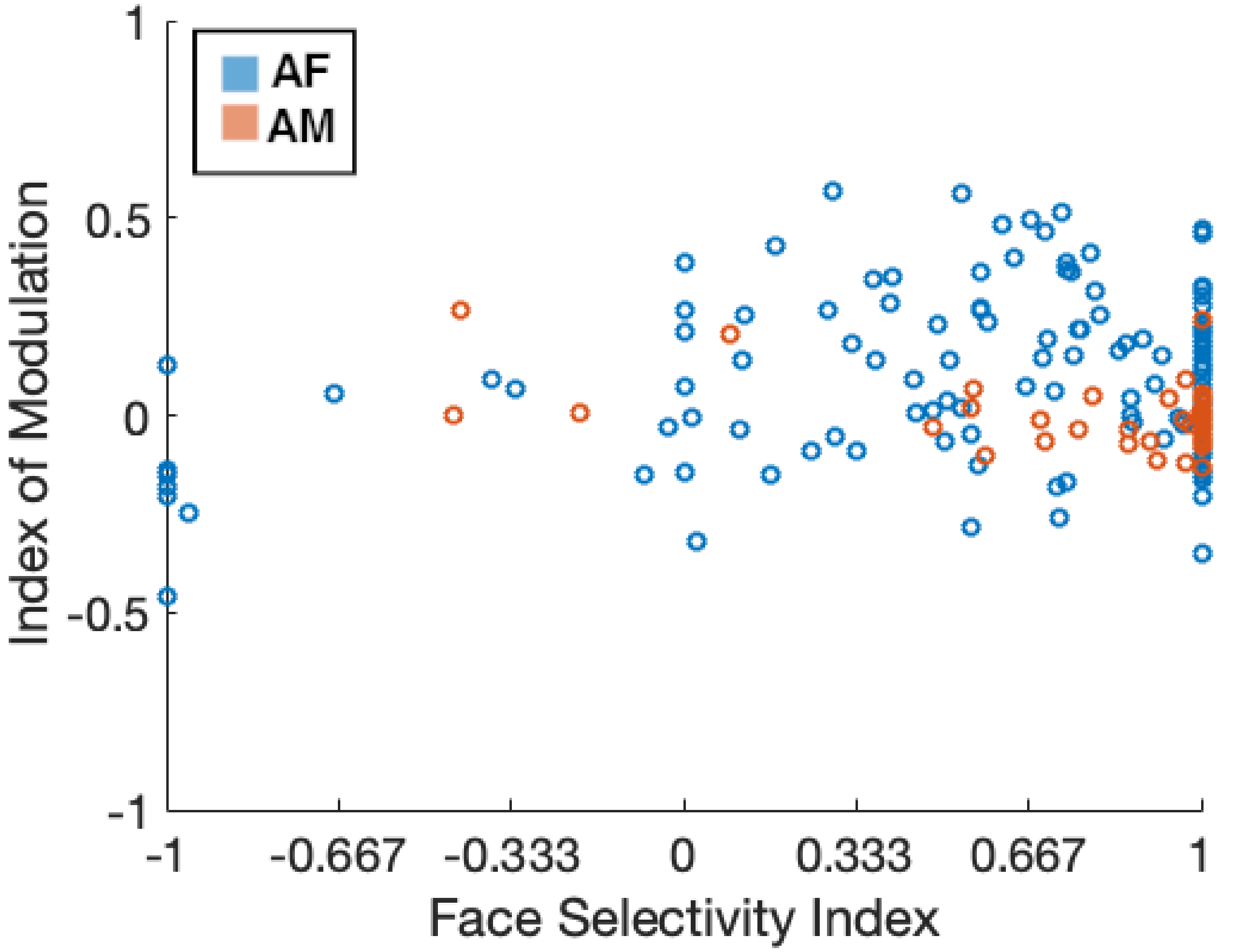

Figure S3. Relationship of Face Selectivity and Audiovisual Modulation related to Figure 3 and Table 1. Scatter plot comparing the face selectivity index with the index of modulation for all AF cells (blue) and AM cells (orange).
